# A Mean Field Poisson–Boltzmann Theory Assessment of Copper Oxide Nanosheets Interaction Potential in Physiological Fluids

**DOI:** 10.3390/nano15171330

**Published:** 2025-08-29

**Authors:** Mumuni Amadu, Nafisat Motunrayo Raheem, Adango Miadonye

**Affiliations:** School of Science & Technology, Cape Breton University, Sydney, NS B1M 1A2, Canada; nafisat_raheem@cbu.ca (N.M.R.); adango_miqdonye@cbu.ca (A.M.)

**Keywords:** Poisson–Boltzmann, zeta potential, surface charge, intracellular, extracellular, electric double layer

## Abstract

In recent times, copper oxide nanosheets (CONSs) have shown a broad spectrum of industrial uses due to their unique properties, including high electrical conductivity, surface-enhanced catalytic activity, etc. Therefore, industrial processes involved in their manufacture can give rise to airborne particulates. Several in vivo studies have reported toxicity of these nanoparticles due to their interactions with biological molecules. Generally, literature-based assessment of their toxicity has centered on experimental findings. In this paper, we report for the first time, trend in CONSs interactions in intracellular and extracellular fluids, using the Nonlinear Mean Field Poisson–Boltzmann theory. Our theoretical prediction for zeta potential in the extracellular fluid environment align with published values in the literature. Based on this theoretical approach, we also demonstrate that double layer disjoining pressure due to interacting double layers of CONSs is generally higher in intracellular fluids. The findings of our theoretical approach highlight the importance of predicting the extent of cellular uptake potential of CONSs in organs that are prone to such airborne environmental particulates.

## 1. Introduction

Recently, there has been a proliferation in the use of copper oxide nanosheets (CONSs) due to their effectiveness in speeding up reactions [1,2,3] and surface coatings, such as on carbon cloth as Binder-Free Electrode for asymmetric supercapacitors [4]. Cu/Cu_2_O nanostructures derived from copper oxalate are promising materials for non-enzymatic glucose oxidation electrocatalysts, offering high sensitivity, selectivity, and stability [5]. Also, in the field of agriculture, copper oxide nanoparticles (CuONPs) have been used as pesticides, herbicides, fertilizers, additives for soil remediation, and growth regulators [6], where both positive and negative impacts have been reported in plants. For instance, Shende et al. [7] have demonstrated the effectiveness of CuONPs in enhancing the growth of pigeon pea legumes. The potential for generation of airborne particulates of CONSs as well as their association with environmental and occupational health hazards is huge [8,9]. From the toxicological viewpoint, nanoparticles (NPs) are receiving increasing attention from many scientific communities and environmentalists due to their strong influence on human health [10]. The environmental presence of such nanoparticles stems from the increased uses of nanotechnology in consumer and other industrial products. In this regard, their ultrafine sizes mean they can be suspended in the atmosphere for a long time, and can thus travel large distances and cause several health issues due to exposure. Inhalation of some airborne nanoparticles [11,12] can have detrimental health effects, particularly on respiratory and cardiovascular systems [13]. Moreover, the small size of nanoparticles allows them to also penetrate cell membranes and interact with cellular components with the potential for DNA damage [14].

Literature shows that the absorption of NPs in the pulmonary system is a possibility [15] as it is in the ocular system [16]. Also, NPs crossing of blood–brain barrier has been proven as a new therapeutic approach to central nervous system diseases [17], and the potential for electrostatic interactions in such organs cannot be ruled out. Also, zeta potential in cellular fluids in such organs is an indirect measure of surface charge, and it is typically measured using electrophoretic light scattering (ELS) [18] where particles move in an electric field and their velocity (electrophoretic mobility) is related to electrokinetic parameters, which can be calculated using theoretical models like the Smoluchowski equation. In the literature, the extent of interaction of NPs with biological matrix/media has been determined using bioassay techniques [19,20,21], which depend on detection and quantification. Moreover, the extent to which detection and quantification of NPs interactions in biological systems as revealed in bioassay will correlate with the extent of their electrostatic interaction. Generally, NPs interact in biological media by electrostatic means [22], such as with charged biological molecules [23]. Copper Oxide NP sheet has a definite isoelectric point [24]. Therefore, at the pH of an aqueous medium, such as intracellular or extracellular fluid, it will develop a unique charge that will warrant the evolution of the Electric Double Layer (EDL) with the potential to electrostatically interact among the layers and with biological molecules.

Fundamental to the underlying electrostatic theory is the formation of the Electric Double Layer (EDL) at physiological pH [23,25]. In the context of the EDL structure, the zeta potential is the potential at the shear plane, with a direct correlation to the surface potential and surface charge density [26]. Considering nanoparticle toxicological potential, Zeta potential has been used as the basis for assessing cellular interaction and uptake. In this regard, Zeta potential is generally used to characterize the charge of NPs and to predict NP toxicity, where, cationic NPs are generally more toxic than anionic ones because of their greater cellular uptake [27,28,29].

Understanding the extent of NPs health hazard depends on understanding the electrostatic interactions. The Mean, Field Poisson–Boltzmann (MFPB) theory, is a nonlinear partial differential equation which describes the spatial variation of the electrostatic potential with distance in the electric double layer (EDL) [30,31,32]. Its solution exists, which links the surface charge density to zeta potential [33,34]. Therefore, given the formation of the electric double layer at the CONSs-biological fluids interface, derivation of surface charge density and zeta potential from the analytical solution to the MFPB theory provides an alternative means for studying the extent of CONSs interactions in intracellular and extracellular fluids. To the best of our knowledge, while experimental based methodology for zeta potential/surface charge assessment for NPs in physiological fluids has been known, the theoretical approach for calculating these parameters specifically for NPs-biological systems, especially for copper oxide CONSs is lacking. Moreover, physiological analysis, for in vitro, and in vivo in research are the two main approaches to assessing nanomaterials’ toxic effects [35]. Thus, the MTT(3-[4,5-dimethylthiazol-2-yl]-2,5 diphenyl tetrazolium bromide) assay method which measures cell viability in cytotoxicity study of copper oxide nanoparticles has been used. It works by detecting the conversion of a water soluble dye into an insoluble counterpart by metabolically active cells, where the amount produced is proportionl to the number of viable cells [36]. Therefore, to fill the knowledge gap, we tackle this task, focusing on the theoretical basis of the MFPB theory as found in colloidal science. We base our research work on CONSs (Copper oxide nanosheets) due to the emerging diverse applications [37,38,39,40]. An extensive body of literature exists for the analytical solution, focusing largely on 1:1 electrolyte (symmetric systems [41,42]. Intra and extracellular fluids contain multivalent ions and their mixtures, limiting the symmetric approach. Moreover, they have low electrolyte concentration, limiting the use of the linearized approach of Debye-Hackle [43]. Analytical solutions exist for multi-valent ion systems [44] and their mixtures as well as for nonlinear cases as opposed to the classical Debye Hackle linearization [45,46], where the potential drop across a double layer is insignificant compared to Boltzmann’s thermal energy, as is the case for high electrolyte concentration. The solution describes the electrostatic potential distribution around charged molecules in biological fluids, accounting for both the potential generated by the molecule and the influence of the surrounding electrolytic medium. Consequently, the model is particularly useful for understanding processes related to ion binding, protein-protein interactions, and the dynamics of biomolecules in physiological fluids [47]. However, there is hardly any report in the literature documenting its theoretical application to intracellular and extracellular fluids related to CONSs. Therefore, in this paper, we exploit the theoretical solution of the nonlinear Poison Boltzmann Equation (PBE) by modeling CONSs as sheet like with distinct surface chemistry, with their surfaces interacting electrostatically with physiological fluids in biological systems, in a manner that is pH dependent [48]. This approach enabled us to seek solutions within the context of the application of the PBE to adsorption on clay minerals [49], which guided our choice of the appropriate nonlinear analytical solution [50,51], that integrates the effects of multi-ion valency, given that biological fluids have mixed electrolytes [52] with concentrations that limit the use of the linearized approach. In physiology, extracellular fluid (ECF) is vital, as it acts as the medium for exchange between cells and the rest of the body, assisting in the delivery of nutrients and removal of waste products. Moreover, it is the environment where cellular processes involving oxygen and carbon dioxide exchange occur [53]. Therefore, the presence of CONSs in such fluids and the resulting electrostatic interaction has the potential to undermine such vital physiological roles. In terms of nanoparticle toxicology, literature distinguishes between neutral and nonneutral particles in light of biological interactions, based on the magnitude of zeta potential. Therefore, in this paper, we focus theoretically on zeta potential and surface charge density of CONSs in the extracellular fluid environment. We extend the nature of electrostatic interactions, by calculating EDL disjoining pressure forces for the lungs, brain, and cornea regions in both extracellular and intracellular fluids, given the greater potential of these organs’ exposure to nanoparticles [54]. We develop a robust methodology based on the theoretical foundations of the analytical solution of the PBE that required us to calculate critical parameters of intracellular and extracellular fluids, namely static dielectric constant and Debye length needed for theoretical calculations of surface charge, zeta potential and double layer disjoining pressure. Finally, we discuss the theoretical results considering trends in electrostatic interaction of CONSs in intracellular and extracellular fluids found in the lung, brain, and cornea. We have demonstrated theoretically that double layer disjoining pressure is generally higher in intracellular fluid than in extracellular fluids for all the organs studied in addition to also showing the capability of the PBE to produce theoretical results that match experimentally measured ones. Our findings are useful for gaining an insight into CONSs interactions potential in such systems, which can have toxicological dimensions. The novelty of our study stems from the fact that the PBE has been used to theoretically predict electrostatic interactions of CONSs in intracellular and extracellular fluids, which supplements studies in such systems based solely on experimental approaches.

## 2. Backgrounds

**Figure 1:**
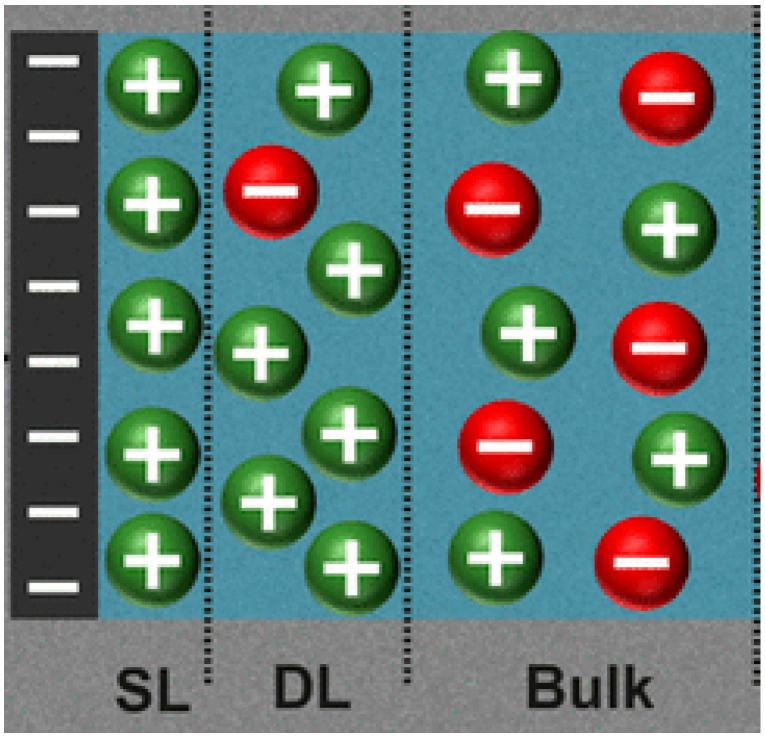
Electric double layer structure at the copper oxide nanosheet-aqueous fluid interface (green color for cations and red for anions) [57].

At a metal oxide-water interface, silanols (SiOH) are formed through a combination of hydration and hydrolysis reactions [55]. These amphoteric surface ionizable groups deprotonate or protonate in accordance with the aqueous solution interface to develop surface charges [56]. Therefore, at the copper oxide nanosheet-physiological fluid interface, redistribution of intra and extracellular fluid ions will occur in response to the emerged surface charge, a mechanism which prompts the evolution of the EDL, structurally identified as the stern layer (SL), diffuse layer (DL), and the bulk solution [57] (See Figure 1).

The deprotonation and interfacial surface complexation reactions can be described, using the following thermodynamic approach [58]:

(1) $$\equiv SOH_{2}^{+}↔\equiv SOH+H^{+}$$

(2) $$\equiv SOH↔\equiv SO^{-}+H^{+}$$

(3) $$\equiv SOH_{2}^{+}An↔\equiv SOH+An+H^{+}$$

(4) $$\equiv SOH+Cn↔\equiv SO^{-}Cn^{+}+H^{+}$$

(5) $$K_{a_{1}}=\frac{\left[H^{+}\right]\left[\equiv SOH\right]}{\left[\equiv SOH_{2}^{+}\right]}\frac{\gamma_{H}\gamma_{0}}{\gamma_{\pm }}exp\left(\frac{-e\psi_{0}}{kT}\right)$$

(6) $$K_{a_{2}}=\frac{\left[H^{+}\right]\left[\equiv SO^{-}\right]}{\left[\equiv SOH\right]}\frac{\gamma_{H}\gamma_{-}}{\gamma_{0}}exp\left(\frac{-e\psi_{0}}{kT}\right)$$

(7) $$K_{A_{n^{-}}}=\frac{\left[H^{+}\right]\left[An^{-}\right]\left[\equiv SOH\right]}{\left[\equiv SOH_{2}^{+}An^{-}\right]}\frac{\gamma_{H}{\gamma_{An}\gamma }_{-}}{\gamma_{0}}exp\left(\frac{-e\left(\psi_{0}-\psi_{\beta }\right)}{kT}\right)$$

(8) $$K_{{C_{n}}^{+}}=\frac{\left[H^{+}\right]\left[SO^{-}Cn^{+}\right]\left[\equiv SOH\right]}{\left[\equiv SOH\right]\left[Cn^{+}\right]}\frac{\gamma_{H}\gamma_{\gamma_{\pm }}}{\gamma_{0}}exp\left(\frac{-e\left(\psi_{0}-\psi_{\beta }\right)}{kT}\right)$$

where, $K_{a_{1}}$ is the dissociation constant of surface ionizable group $\equiv SOH_{2}^{+}$ [M], $\;K_{a_{2}}$ is the dissociation constant of surface ionizable group $K_{a_{2}}$ [M], $\;K_{A_{n^{-}}}$ is the anion complexation constant [M^2^], $K_{{C_{n}}^{+}}$ is cation complexation constant [M^2^],$\;\psi_{0}$ is the surface potential [V], $\psi_{\beta }$ is the potential of the inner Helmholtz plane [V], $\gamma_{H}$ is the activity coefficient of the hydrogen ion concentration $\;\left[H^{+}\right]$, $\gamma_{0}$ is the activity coefficient of the ≡SOH groups [-], $\gamma_{+}$ is the activity coefficient of $\equiv SOH_{2}^{+}$ groups [-], $\gamma_{-}$ is the activity coefficient of the $\equiv SO^{-}$ groups [-], $\gamma_{An}$ is the activity coefficient of the anion anion [-], $\gamma_{Cn}$ is the activity coefficient of the cation [-], $\gamma_{\pm }$ is the activity coefficient of the $\equiv SOH_{2}^{+}An^{-}$ groups [-], and $\gamma_{-,+}$ is the activity coefficient of the $SO^{-}Cn^{+}$ groups [-].

Fundamentally, the mechanisms involved in the formation of the EDL, which comprises ionization of surface ionizable groups and the resulting redistribution of interfacial aqueous solution ions to shield the electrostatic surface charge density is well described by the Mean Field Poisson–Boltzmann theory. Accordingly, the thermodynamic basis provides a solid foundation for studying the electrokinetics parameters involving zeta potential, surface charge density, and interaction potentials, which form the basis of the theoretical framework required to achieve the objective of this paper, and the following sections will be devoted to the discussion of fundamental theories.

## 3. Theoretical Foundation

### 3.1. Mean Field Electrostatic Theory

#### 3.1.1. Zeta Potential Model

The salinity of physiological fluids is generally low [59], warranting the solution of the Poisson–Boltzmann equation in its nonlinear form [60,61]. In this paper, we model copper oxide, nanoplatelets as planar substrates like graphene ones [62,63], to exploit the context of the electric double layer structure. The electrostatic field potential gradient is given as [50,64]:

(9) $$\frac{dy}{dx}=-sgn\sqrt{\frac{8F^{2}}{\varepsilon_{0}\varepsilon_{0}T}\sum_{i}\left(f_{i}exp\left(-Zy\right)-1\right)}$$

In a 1:1 and 2:1 electrolyte mixture, the solution to Equation (1) is given as [64]:

(10) $$\psi \left(x\right)=\frac{RT}{F}ln\left[\left(\frac{f_{A}-3f_{C}}{f_{A}-2f_{C}}\right)\left(\frac{1-\lambda_{1}exp\left(-Κx\right)}{1+\lambda_{1}exp\left(-Κx\right)}\right)-\frac{f_{C}}{f_{A}+2f_{C}}\right]$$

where,

(11) $$\lambda_{1}=\frac{\sqrt{f_{A}+3f_{c}}-\sqrt{\left(f_{A}+2f_{c}\right)e^{y_{0}}+f_{c}}}{\sqrt{f_{A}+3f_{c}}+\sqrt{\left(f_{A}+2f_{c}\right)e^{y_{0}}+f_{c}}}$$

(12) $$y_{0}=\frac{F\psi_{0}}{RT}$$

where, $y_{0}$ is the dimensionless surface potential [V], *F* is Faraday’s constant [Cmol^−1^], $\psi_{0}$ is the surface potential [V], R is then universal gas constant [JK^−1^ mol^−1^], and *T* is the absolute temperature.

(13) $${Κ}^{2}=2e^{2}n_{b}/\varepsilon k_{B}T$$

where Κ is the Debye screening length [m], e is the electronic charge [C], $n_{b}$ is the number density of ions in solution [m^−3^], ε is the permittivity [Fm^−1^], and $k_{B}$ is Boltzmann’s constant [JK^−1^].

#### 3.1.2. Zeta Potential Dependence on pH

Since protons and hydroxides ions are the potential determining ions for oxides, hydroxides, and silicates in water, the following can be written for the surface potential $\psi_{0}$ for CONSs [65,66].

(14) $$\psi_{0}=-2.303\frac{K_{B}T}{e}\Delta pH=2.303\frac{K_{B}T}{e}\left({pH}_{pzc}-pH\right)$$

In Equation (14), ${pH}_{pzc}$ is the point of zero charge pH of surface of CONS.

Thus, Equation (14) can be written as:

(15) $$2.303\frac{K_{B}T}{e}\left({pH}_{pzc}-pH\right)$$

Consequently, combining Equation (15) will facilitate calculation of reduced potential as a function of pH.

#### 3.1.3. Surface Charge Density Model

The link between zeta potential and surface charge density has been studied through electrokinetic and titrimetric methods [67], suggesting an intimate link between the two EDL parameters. The case of a planar geometry, the analytical expression of the surface charge density reads [68]:

(16) $$\sigma_{s}=-\varepsilon \varepsilon_{0}\frac{\partial \psi \left(r\right)}{\partial r}{@}_{x=0}$$

Following Equation (16), differentiating the potential equation (Equation (10)) with respect to *x* and substituting x equal to zero gives:

(17) $$\sigma =\varepsilon_{0}\varepsilon_{r\;}A\left(\frac{BCD}{1+C}+\frac{B(1-C}{{\left(1+C\right)}^{2}}\right)C*{\left(\frac{B*\left(1-C\right)}{1+C}-E\right)}^{-1}$$

In Equation (17), A equals $\frac{RT}{F}$, B equals $\left(\frac{f_{A}-3f_{C}}{f_{A}-2f_{C}}\right)$. C equals $\lambda_{1}$, D equals Κ, and E equals $\frac{f_{C}}{f_{A}+2f_{C}}$.

Equation (16) can be used to calculate surface charge density, given the value of zeta potential calculated from Equation (10) in light of mixed electrolyte systems, where the distance *x*, represents the distance to the hydrodynamic shear plane.

#### 3.1.4. Double Layer Repulsion Model

Double layer forces between charged objects exist across liquids, typically in aqueous media, acting over distances that are comparable to the Debye screening length, with their magnitude increasing with surface charge density [69].The double layer interaction for interfaces having different potentials $\zeta_{1}$ and $\zeta_{2}$, can be approximated under the constant potential approximation theory, which gives [70]:

(18) $$F_{D}\left(D\right)=n_{b}k_{B}T\left\{\left[2y_{1}y_{1}cosh\left(Κh\right)-y_{1}^{2}-y_{2}^{2}\right]/{\left(sinh\left(Κh\right)\right)}^{2\;}\right\}$$

In Equation (18) $y_{1}$ and $y_{2}$ are reduced potentials defined as:

(19) $$y_{1}=\frac{F\psi_{01}}{RT}$$

(20) $$y_{2}=\frac{F\psi_{02}}{RT}$$

where, $\psi_{01}$ and $\psi_{02}$ are the surface potentials of respective electric double layers.

Equations (2), (15) and (17)–(20) form the fundamental theoretical foundations of the present paper and in Section 4, we will discuss their uses regarding the principal goal of the paper.

## 4. Methodology

### 4.1. Dielectric Permittivity

More than 60% of our body substance consists essentially of fluids [71] containing various ions and substances dissolved or suspended in aqueous media, such as intracellular (K^+^ dominated) and extracellular (Na^+^ dominated) in origin [72]. Knowledge of the exact concentrations of ions in the body is needed to maintain essential physiological processes involving muscular contraction, nerve excitation, and neural impulse conduction [73]. The ionic composition of the body is quantified by calculating molarity and expressing it in millimoles (mM) per liter. Table 1 shows the concentration of different ions in intercellular and extracellular fluids.

Equation (16) for calculation of surface charge density requires knowledge of the static dielectric permittivity of the aqueous medium, which is calculated as [75]:

(21) $$\varepsilon_{0}\left(T,N\right)=\varepsilon_{0}\left(T,0\right)a\left(N\right)$$

(22) $$a\left(N\right)=1.000-0.255N+5.15\times 10^{-2}N^{2}-6.889\times 10^{-3}N^{3}$$

where, N is the normality.

(23) $$N=S\left(1.707\times 10^{-10}+1.205\times 10^{-5}S+4.058\times 10^{-9}S^{2}\right)$$

where, S is the salinity in parts per thousand for 0≤S≤260

(24) $$\varepsilon_{0}\left(T,0\right)=87.74-0.40008T+9.398\times 10^{-4}T^{2}+1.410\times 10^{-6}T^{3}$$

where, T is the temperature in Celsius.

### 4.2. Debye Length

Debye length is a fundamental length scale that is salinity dependent. It is a measure of a charge carrier’s net electrostatic effect in a solution and how far its electrostatic effect persists [76]. Its calculation requires knowledge of the ionic strength of the electrolyte. The ionic strength is calculated as [77]:

(25) $$I=0.5\sum_{1}^{n}cz^{2}$$

where, I, represents the ionic strength [M], c is the concentration of ion [M], and z is the ionic valence.

From Table 1, and for simplicity, we assume extracellular fluid is a predominant mixture of sodium chloride and calcium bicarbonate ($NaCL+Ca{\left(HCO_{3}\right)}_{2}$. Therefore, the ionic combinations are 1:1 and 2:1, Based on the assumption, the square of the reciprocal of Debye, ${Κ}^{2}$ length is calculated as:

(26) $${Κ}^{2}=2e^{2}n_{b}/\varepsilon k_{B}T$$

In which, $n_{b}$ is the number density of ions [m^−3^] and ε dielectric permittivity [Fm^−1^].

The ionic strength, calculated using Equation (25) was used to calculate the density of ions in Equation (26) The calculated ionic strength was converted to normality for the calculation of the static dielectric permittivity, using Equation (20) through Equation (24). The ionic strengths were calculated as 0.42 M and 0.14 M respectively. Using Equation (20) through Equation (24), the static dielectric constant of intra and extracellular fluids were calculated as 74.25 and 74.28 respectively. Using values of ionic strength, the number density of ions in intra and extracellular fluids were calculated by converting moles per liter to moles per cubic meter and the results were multiplied by Avogadro’s number. The values were 2.6 × 10^26^ and 8.68 × 10^25^ for intracellular and extracellular fluids respectively.

### 4.3. Calculation of Double Layer Repulsion

To theoretically study the extent of electrostatic interaction of CONSs in biological fluids, using Equation (17), we calculated values of the reduced potential using Equation (15), as a function of pH. Gaohua et. al. [78] have published data on the pH of different parts of the body (See Appendix A). We extracted corresponding values of reduced potential, using the corresponding pH of the region where the organ is located. Values of reduced potential and the number density of ions for intra and extracellular fluids were then used to calculate double layer disjoining pressure as a function of CONSs separation based on Equation (18).

### 4.4. Assumption for Zeta Potential Calculations

The zeta potential is the potential at the shear plane [79], corresponding to the shear plane distance when a part of the diffuse layer is transported by fluid flow [80]. The zeta potential can be obtained from Equation (10) as:

(27) $$\psi \left(\zeta \right)=\frac{RT}{F}ln\left[\left(\frac{f_{A}-3f_{C}}{f_{A}-2f_{C}}\right)\left(\frac{1-\lambda_{1}exp\left(-Κx_{sh}\right)}{1-\lambda_{1}exp\left(-Κx_{sh}\right)}\right)-\frac{f_{C}}{f_{A}+2f_{C}}\right]$$

In Equation (27) $x_{sh}$ [m] is the distance to the hydrodynamic plane of shear [81].

Because the position of the shear plane is very close to the surface, a distance of about 2.4 × 10^−10^ m is often used Revil and Glover [82,83]. To calculate zeta potential using Equation (27), we use a value of $x_{sh}$ equal to 2.4 × 10^−10^ m. The shear plane in the electric double layer (EDL) is located at the boundary between the Stern layer and the Diffuse layer, and its distance measured from the charged surface is roughly equivalent to the thickness of the Stern layer. Generally, this distance is of the order of the molecular diameter of water (0.275 nm) [84] and independent of the chemistry of the aqueous fluid. Therefore, the value of the shear plane distance chosen in this paper is suitable for achieving the objective of the study.

In this paper, a temperature of 37 °C corresponding to the normal human body temperature was used for all equations containing temperature, which is equivalent to 310 K [84,85,86]. The approach is consistent with the research work of Van et al. [87] in which cells were grown at 37 °C.

### 4.5. Point of Zero Charge pH of CONS

Several values of copper oxide point of zero charge pH has been reported in the literature, with values depending on the methodology employed [88]. Monoclinic oxides have been frequently used in adsorption studies because of their unique structural properties related to high surface area [89]. Zhao et al. [90] have determined a value of 6.8 for monoclinic copper oxide. Ejeta et al. [91], have determined a value of 7, using the pH drift method, which is highly popular due to the unique experimental feasibility of the method. In this paper, we used an average of the two values, which is 6.9. Values of fundamental physical constants used in this paper are found in Appendix B.

To exploit the theoretical basis of the present paper for achieving the objective of the study, there is the need to address the question of limitations. Therefore, to avoid any limitations regarding the choice of planar CONSs, we consider the case of an infinitely extended planar surface, where there are two dimensions in which the potential cannot change because of symmetry. Assuming these dimensions are the y and z dimensions, only the x dimension is left [92]. Also, to address the question of chemical heterogeneity of the substrate, we assume that CONSs are synthesized through various methods, including hydrothermal synthesis, green synthesis using plant extracts, and other solution-based approaches. These approaches guarantee surface smoothness and homogeneity as well [93].

## 5. Results and Discussion

The wide use of copper oxide nanoparticles has necessitated studies to understand their toxicological impact in biological systems, such as, bacterial, algae, fish, rats, human cell lines etc. where key factors, such as particle shape and size play major roles [94]. The following sections are devoted to discussing the theoretical findings of this paper, considering the potential for cellular uptake of CONSs, the related electrostatic interactions, as well as possible clinical implications.

### 5.1. Theoretical Zeta Potential Characteristics of Extracellular Fluids

**Figure 2:**
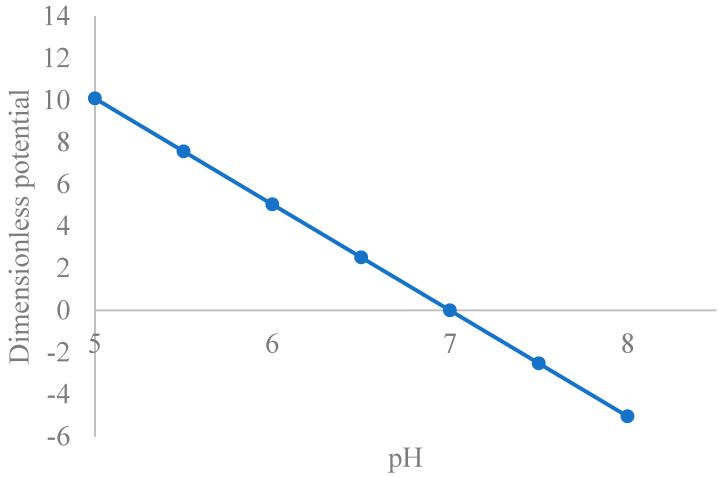
A plot of reduced surface potential vs. pH for CONS.

**Figure 3:**
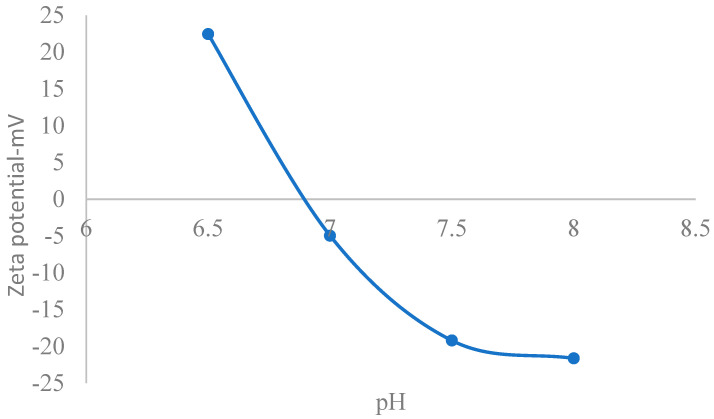
A plot of zeta potential vs. pH for CONS in the extracellular fluid environment.

Figure 2 shows a plot of reduced surface potential versus pH, with decreasing values as pH increases towards the point of zero charge pH (6.9). In physiological fluids, CuO nanoparticles typically exhibit a negative zeta potential due to the dissociation of surface hydroxyl groups, resulting in a charged surface, the magnitude of which indicates the strength of the electrostatic repulsion between nanoparticles [95]. Consequently, an analytical solution to the PBE within physiological pH must reflect the trend reported by the cited reference. Intracellular fluid (ICF) is vital for diverse physiological processes including cellular function and overall health [96]. In this regard, its zeta potential was studied. Accordingly, Figure 3 shows that the zeta potential is averagely negative, decreasing with pH [97]. The figure shows that within physiological pH (closer to neutral), CONSs are negatively charged. A zeta potential of −16.6 mV has been reported in previous studies [98,99], while values of −8 mV and −9 mV have also been reported in relationship to toxicity studies [100] which aligns with that obtained in the present study. Moschini, et al. [101], have also reported values of −8.6 mV and −9.6 mV in cells cultures, which align with Figure 3. Semisch et al. [102], have also reported zeta potential for copper oxide in biological fluid ranging from 13.1 mV to −14.4 mV, which also fall within the range in this paper.

**Figure 4:**
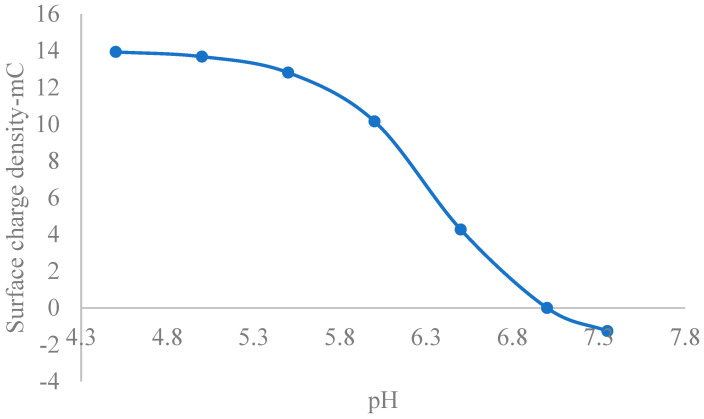
A plot of surface charge density of CONSs in the extracellular fluid environment.

Figure 4 shows a plot of surface charge density vs. pH, with decreasing value. Within the physiological pH regime, many biological molecules, including proteins, nucleic acids, and some lipids, carry a net negative charge due to the deprotonation reactions of acidic functional groups like carboxyl groups (-COOH), which become negatively charged (COO-) under these conditions [103]. Electrostatic repulsion between CONSs and negatively charged biological species in the extracellular environment is imminent. Recently, Weiss et al. [104] demonstrated that the surface charge of cationic nanoparticles is a better measure of toxicity than zeta potential. Therefore, given the averagely negatively charged nature of CONSs, which correlates with zeta potentials electrostatic repulsion is imminent. In the literature, values of zeta potential for CONPs have been reported, but data on surface charge density are lacking. However, given the direct relationship between the two parameters, the moderately negative lower values of surface charge density within the physiological pH range suggest weak electrostatic interactions with negatively charged biological molecules.

Evidence abounds in the literature that copper oxide nanoparticles (CuONPs) exposure to living systems cause generation of reactive oxygen species, oxidative stress, inflammation, cytotoxicity, genotoxicity and immunotoxicity [102,105,106]. Generally, cellular uptake of nanoparticles becomes possible where there is electrostatic attraction due to the presence of oppositely charged biological species in cellular environments. Moreover, based on toxicological classification, nanoparticles with a zeta potential between −10 and +10 mV are considered approximately neutral, as opposed to greater than +30 mV or less than −30 mV, which are considered strongly cationic and strongly anionic, respectively [107]. Accordingly, in Figure 3, at pH of 7.5 (physiological pH), the zeta potential is −19 mV, testifying to the strongly anionic character of CONS in the extracellular fluid environments. This theoretical finding for the extracellular fluid environment aligns with the findings of Shao et al. [108], who studied cytotoxicity of nanoparticles to cells using MTT (3-(4,5-dimethyl-2-thiazolyl)-2,5-diphenyl-2-H-tetrazolium bromide) assay, demonstrating that a zeta potential of ±10 millivolts (mV) is generally considered to be a neutral or low-charged surface on nanoparticles or colloids, and is associated with less cell wall destruction and lower toxicity than particles with high zeta potentials. Midekessa, et al. [109], have reported a mean value of −24 mV in relationship to measurement in extracellular vesicles, which is within the range found in Figure 3. Moschini, et al. [101], have also reported values of −8.6 mV and −9.6 mV in cells cultures, which align with Figure 3. Semisch et al. [102], have reported a zeta potential for copper oxide in biological fluid ranging from 13.1 mV to −14.4 mV, which also falls within the range this paper.

The thickness of the Stern layer, which is a region in the electrical double layer near a charged surface, has an inverse relationship with zeta potential. As the Stern layer thickness decreases due to increased electrolyte concentration, the zeta potential generally decreases as well. This is because a thinner Stern layer results in higher surface charge density, which in turn leads to a smaller zeta potential [110]. However, considering that physiological fluids generally have low electrolyte concentration, the sensitivity of zeta potential to the stern layer thickness was neglected in this study.

### 5.2. Stability of CONSs in Extracellular Fluids

As a general rule, values of zeta potential −30 mV and ≤−60 mV is considered good with excellent colloidal stability, respectively [111,112], while zeta potential slightly below −30 mV indicates monodispersing without aggregates [113]. On the other hand, zeta potential approximately equal to −20 mV is an indication of short-term colloidal stability with a tendency for rapid aggregation [112]. Therefore, theoretically, the values of zeta potential in intracellular fluids within physiological pH implies short-term stability.

**Figure 5:**
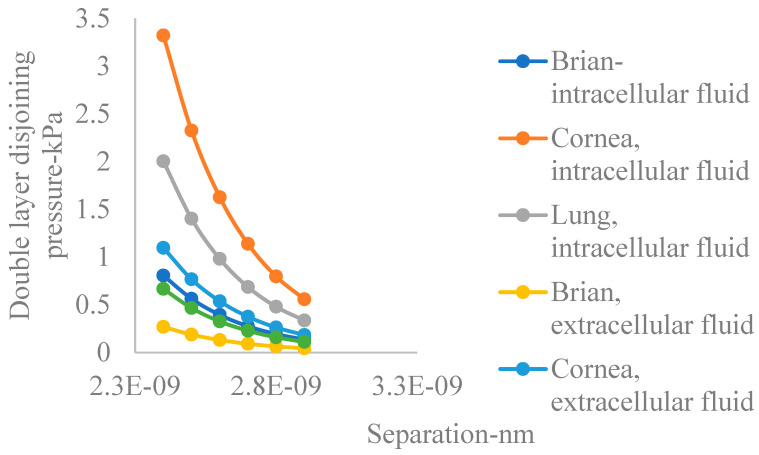
Double layer disjoining pressure for CONSs in different regions of the body.

Airborne particulate copper oxide nanoparticles released from processing plants are cause for environmental and health concerns due to their high surface area and reactivity in biological systems [94]. In physiological fluids characterized by mean ambient pH below the mean point of zero charge pH, the evolution of the electric double layer implies imminent interactions among electric double layers. The lung, cornea, and brain are particularly susceptible to the damaging effects of environmental pollution [54]. Figure 5 shows plots of double layer disjoining pressure with distance of separation for the different portions of the human body in both intra and extracellular fluids, based on the number density of ions calculated in this paper. The figure reveals the following information contained in Table 2.

Accordingly, double layer disjoining pressure is generally higher in intercellular fluids compared to extracellular fluids. Also, the figure shows that on the average, in intracellular fluids, the cornea has the highest double layer disjoining pressure, followed by the lung, with the brain having the lowest. This trend also applies in the case of extracellular fluids.

When nanoparticles (NPs) enter biological systems, they rapidly become coated with proteins, forming nanoparticle-protein corona [114]. Generally, a greater surface charge density on a nanoparticle leads to a thicker and denser protein corona, as the stronger electrostatic interactions attract more proteins to the NP surface [115]. Considering the trend in electrostatic interactions revealed moderate values of zeta potential and surface charge density so that nanoparticle-protein corona formation will occur to a moderate extent in extracellular fluids.

### 5.3. Cellular Uptake

The impact of CuO NP on the DNA damage response on the transcriptional level has been studied by the quantitative gene expression profiling technique. The study analyzed the cytotoxicity, copper oxide uptake and the impact on the oxidative stress response, cell cycle regulation and apoptosis, revealing cellular concentrations at toxic levels [116]. In another study, copper oxide nanoparticles (CuONPs) exhibited toxic effects in cellular environments due to the release of dissolved copper ions (Cu^2+^), which have the potential to induce oxidative stress, leading to cell damage and apoptosis [117]. Considering the theoretical findings of the present paper and considering the fact that at physiological pH values most cellular components are negatively charged, direct cellular uptake of CONSs in the extracellular fluid environment is not possible due to the imminent electrostatic repulsion. Therefore, the documented effect of copper oxide nanoparticles as found in the above cited literature is only possible through two mechanisms, namely selective absorption of proteins and charge reversal [118] and dissolution of copper oxide and subsequent release of copper two ions into cells [119]. For instance, negatively charged copper oxide (Cu_2_O) ions can selectively adsorb proteins due to electrostatic attraction and the ability of the oxide surface to form complexes with protein molecules, where the negative charge on the Cu_2_O surface attracts positively charged sites on protein molecules, leading to binding [120]. Another option is the interaction with sulfur containing groups of proteins [121], leading to stabilization. According to Aggarwal et al. [122], the most adsorbed proteins detected on nearly all NPs are those that are highly abundant in the blood, which were albumin, immunoglobulin (IgG), fibrinogen and apolipoproteins. Such selective absorption can cause charge reversal of negatively charged CONSs to cause their cellular uptake by negatively charged biological molecules like cell membranes, leading to cellular damage. On the possibility of cellular uptake of dissolved copper ions from copper oxide nanoparticles dissolution, Boyadzhiev et al. [117], have already demonstrated it through invivo studies. Thus, although values of zeta potential between ± 30 mV are considered less reactive/toxic, the above outlined mechanism can be responsible for cellular damage in the extracellular environments, which are applicable to CONSs, based on calculated values of zeta potential.

### 5.4. Implications of the Study for Bioassay

The isoelectric point of intracellular fluid proteins is the pH at which the net charge is zero, generally ranging from about 4.0 to 7.0 [123], with many proteins having a value within the range of 5.0 to 8.0. Consequently, most proteins are negatively charged in biological fluids under physiological conditions defined by near neutral pH [124,125]. A major consequence of protein adsorption on nanoparticles is related to size increase in addition to the creation of a predictably negative surface charge density owing to the negatively charged character of most proteins in biological fluids [126,127]. In the era of Nano Precision Medicine, the idea is to design nanoparticles that overcome heterogeneous barriers [128], most importantly, the negative effect of the protein nanoparticle corona effect. The extent of corona formation, its composition and its amount have great implications for any use of nanomedicine. In this regard, nanoparticles with a high tendency for agglomeration upon exposure to proteins, should not be used for the design of target drug delivery. In the context of the theoretical findings of this paper, CONSs in intracellular fluids will have less electrostatic interaction. Therefore, in areas of CONPs application in bioassay for extracellular fluid protein characterization [129], the low values of negatively surface charged density imply limited cellular fluid-protein adsorption, which underscores its limited application.

### 5.5. Clinical Implications for the Theoretical Findings

The technological use, environmental circulation [130,131] and toxicity [132,133] of nanoparticles derive from their surface chemistry, charge and state of aggregation, which underscore their proper characterization for a particular application. In the literature, the toxicological profile of copper oxide nanoparticles based on in vivo studies has been greatly limited nonhuman [134,135,136,137], with studies to humans being limited human cell models [138].

Literature shows that the increasing surface charge density of nanoparticles increases the rate of dissolution [139,140]. Therefore, for nanoparticles, due to the increased surface/mass ratio, the specific amount of surface charge (related to the unit mass of solid) increases gradually with decreasing particle size. The implication of the theoretical findings of the present study is that considering the averagely lower zeta potential values of CONSs in extracellular fluids, which translates to averagely lower surface charge density, the dissolution-related cytotoxicity can be minimized by using larger nanoparticles. This strategy will be clinically beneficial for the anticipated use of copper oxide nanoparticle for antimicrobial and anticancer trials in humans [141].

## 6. Summary

In a biological medium, NPs may interact with biomolecules such as proteins, nucleic acids, lipids and even biological metabolites due to their nano-size and large surface-to-mass ratio to form nanoparticle-protein corona (NP-PC) [142]. Overall, the NP-PC can influence the biological reactivity of the NP [143,144]. For instance, nanoparticles can wrap epithelial cell membranes and relocate them across the epithelial cell layer, which can freely diffuse across the damaged epithelial cell layer to relocate cell membrane parts over epithelial layer [145]. Titanium dioxide and calcium carbonate nano particles are known to be biologically toxic due to distinct surface electrokinetic properties. The point of zero charge pH (pzcpH) is the pH of a solid surface at which the net charge is zero [146]. Below the point of pH (pzcpH), the surface develops positive charge and vice versa. In this regard, the pzcpH of CONP is 6.9, and the pH of physiological fluids is found to be in the range of 7.3–7.5 [147], implying an imminent negative charge development in the extracellular fluid domain. The implication is that the negatively charged NPs will electrostatically interact with biological cells/membranes that develop opposite charges, which highlights the toxicity of positively charged NPs to biological components. The Poisson–Boltzmann equation has been applied in a variety of fields, mainly as a modeling tool for approximations relating to charged biomolecular interactions and dynamics of electrons in semiconductors or plasma, among others. In most of the applications, it is used as a model to gain further insight into electrostatics. For instance, Gray et al. [148] used the Poisson–Boltzmann Equation to estimate the electrostatic free energy barrier for dielectric models of biological ion channels. Amadu and Miadonye [149] applied the theory to contact angle problems in the carbon dioxide-brine-solid system. Fixman [150], has demonstrated that the solution to the Poisson–Boltzmann equation is a good approximation for predicting the electrostatics of polyelectrolytes in a biological system. In the present paper, we have used its theoretical foundations to theoretically predict the electrostatics of CONHs in biological systems, demonstrating calculation of zeta potential values that have been reported in the literature. We specifically included the lung in the organs we studied because exposure of CuO NPs (50 nm) to pulmonary epithelial cells of rat systems has been reported to induce concentration dependent DNA damage, caused by lipid peroxidation in addition to oxidative stress [151]. The nanoparticles induced cytotoxicity by generating oxidative stress, cell cycle arrest, and apoptosis [152]. The surface chemistry of CuO is characterized by a variable point of zero charged pH, depending on its source [88]. In this study, we used 6.9.

## 7. Conclusions

In this paper, we have used the analytical solution to the nonlinear Mean Field Poisson–Boltzmann Equation to study theoretically, the electrostatic interactions of similar EDLs for CONSs in the extracellular fluid environment, assuming that the isoelectric point of CuO is closer to 6.9. Calculated parameters are zeta potential and surface charge density, given the direct correlation between these two electrostatic parameters.

1. Based on the NLMFPB electrostatic theory assessment of CONSs in the intracellular fluid environment, they have negative values of zeta potential under physiological pH regimes,
2. Values of zeta potential under physiological pH implies less electrostatic repulsion, which translates to short term stability of CONSs in physiological fluids,
3. Comparing the analytical predictions of zeta potential within physiological pH values obtained in this study to those in literature related to toxicity, CONSs will be non-toxic from the point of view of electrostatic interactions only,
4. Electric double layer disjoining pressure is generally higher in intercellular fluids compared to extracellular fluids for the organs studied.
5. On the average, and within intracellular fluids, the lung has the highest double layer disjoining pressure, followed by the brain, with the cornea having the lowest.
6. In intracellular fluids, the cornea has the highest double layer disjoining pressure, followed by the lung, with the brain having the lowest.
7. The trend revealed by conclusion number 4 also applies in the case of extracellular fluid.

## Figures and Tables

**Table 1:** Concentration of ions in bodily fluids [74].

Ion	Intracellular Concentration—mM/L	Extracellular Concentration—mM/L
Sodium	10	142
Potassium	149	4
Calcium	0.0001	2.4
Chloride	5	103
Magnesium	58	1.2
Bicarbonate	10	28
Phosphate	75	4

**Table 2:** Trend in double layer disjoining pressure in physiological fluids in the different organs.

Organ	Intracellular	Extracellular
Lung	High	Lower
Brain	High	Lower
Cornea	High	Lower

**Figure A1:**
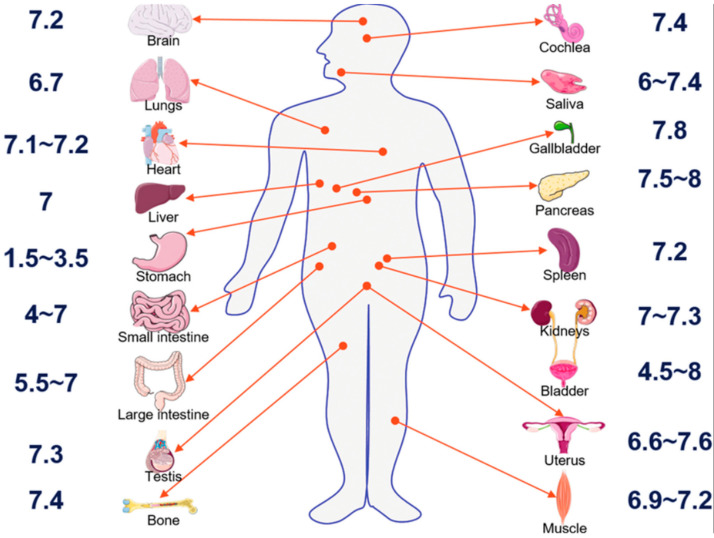
pH of Different Parts of the Human Body [78].

## Data Availability

The original contributions presented in this study are included in the article. Further inquiries can be directed to the corresponding author.

## Abbreviations

The following abbreviations are used in this manuscript:

PBE	Poisson–Boltzmann Equation
MFPB	Mean Field Poisson–Boltzmann
NMFPB	Nonlinear Mean Field Poisson–Boltzmann
CONSs	Copper Oxide Nanosheets
EDL	Electric Double Layer

## Appendix B

**Table A1:** Value of physical constants used in the research work [153].

	Constsnt	Value	Unit
kb	Boltzmann constant	1.38 × 10^−23^	JK^−1^
e	Electronic charge	1.602 × 10^−19^	C
E0	permitivity of vacuo	8.854 × 10^−12^	Fm^−1^
NA	Avogadro’s number	6.022 × 10^23^	Mol^−1^
